# Nomogram for predicting early hypophosphatemia in term infants

**DOI:** 10.1186/s12887-024-04737-8

**Published:** 2024-04-16

**Authors:** Wan Tao, Shina Zhan, Yingjie Shen, Tianjiao Zhao, Feitian Li, Miao Gao, Tingting Yang, Jinqian Yu

**Affiliations:** Neonatal Center, Shunyi Maternal and Children’s Hospital of Beijing Children’s Hospital, No.1 Shunkang Road, Shunyi District Beijing, Beijing, 101300 China

**Keywords:** Hypophosphatemia, Nomogram, Male, Birth weight, Maternal diabetes, Cesarean delivery, Serum magnesium

## Abstract

**Background:**

Physiological processes rely on phosphate, which is an essential component of adenosine triphosphate (ATP). Hypophosphatasia can affect nearly every organ system in the body. It is crucial to monitor newborns with risk factors for hypophosphatemia and provide them with the proper supplements. We aimed to evaluate the risk factors and develop a nomogram for early hypophosphatemia in term infants.

**Methods:**

We conducted a retrospective study involving 416 term infants measured serum phosphorus within three days of birth. The study included 82 term infants with hypophosphatemia (HP group) and 334 term infants without hypophosphatemia (NHP group). We collected data on the characteristics of mothers, newborn babies, and childbirth. Furthermore, univariate and multivariate logistic regression analyses were performed to identify independent risk factors for hypophosphatemia in term infants, and a nomogram was developed and validated based on the final independent risk factors.

**Results:**

According to our analysis, the multivariate logistic regression analysis showed that male, maternal diabetes, cesarean delivery, lower serum magnesium, and lower birth weight were independent risk factors for early hypophosphatemia in term infants. In addition, the C-index of the developed nomogram was 0.732 (95% CI = 0.668–0.796). Moreover, the calibration curve indicated good consistency between the hypophosphatemia diagnosis and the predicted probability, and a decision curve analysis (DCA) confirmed the clinical utility of the nomogram.

**Conclusions:**

The analysis revealed that we successfully developed and validated a nomogram for predicting early hypophosphatemia in term infants.

## Background

Phosphate, present in hydroxyapatite bone structure, ATP, creatine phosphate, nucleic acids, nucleoproteins, phosphorylation of proteins, 2,3-Diphosphoglycerate, and inorganic phosphate acid-base buffer [1, 2], is crucial for many bodily functions. Many physiological pathways require phosphate, including skeletal development and mineralization, membrane composition, nucleotide structure, cellular signaling, energy storage, and acid-base balance [3, 4]. Nutritional deficiencies, refeeding syndrome, insulin therapy for diabetic ketoacidosis, increased catecholamine secretion, acute respiratory alkalosis, diuretics, glucocorticoids, and antacids are all known to be associated with hypophosphatemia [3, 5–16]. Hypophosphatasia is common in adult and pediatric intensive care units (ICUs). It appears that hypophosphatasia can affect nearly every organ system in the body [16]. For example, hypophosphatasia decreases the contractility of the heart and respiratory muscles [8].

Consequently, when you are suffering from hypophosphates, you are more likely to be at risk of respiratory failure, acute heart failure, cardiac arrhythmias [17], drowsiness, unconsciousness, muscle weakness [9], and cardiac arrest. It also can result in metabolic encephalopathy, proximal myopathy, paralysis, intestinal obstruction, immune dysfunction, hypercalciuria, increased erythrocyte osmotic fragility, insulin resistance, metabolic acidosis, and hemodynamic disorders [1, 5]. The duration of ventilation and the stay in the ICU was extended in hypophosphatemia patients, and they were more likely to develop bronchopulmonary dysplasia [13, 18, 19]. Hypophosphatemia does not always correlate with the severity of clinical symptoms. Most patients with hypophosphatemia may already have potentially fatal complications without clinical symptoms. Maintaining normal phosphate concentrations in the ICU is essential, but phosphate monitoring hasn’t received much attention.

Moreover, if hypophosphatemia is not treated promptly in the early postnatal period, it may lead to metabolic bone disease. Currently, there is no consensus on its definition. It is recommended that cord blood serum phosphate be maintained above 1.5mmol/L, 1.60mmol/L, 1.8mmol/L, 1.9mmol/L, etc [20, 21]. The diagnostic criteria for hypophosphatemia in premature neonates is different from those in full-term newborns. The study aims to identify the association between hypophosphatemia and clinical outcomes among term infants. Hypophosphatemia is defined as less than 1.60 mmol/L in this study. When phosphorus is supplemented, it is quickly absorbed, and virtually no side effects are observed. In this regard, it is vital to monitor newborns with factors that increase their risk of hypophosphatemia and provide them with the appropriate supplements. A retrospective analysis of 82 hypophosphatemia cases in term infants and 334 non-hypophosphatemia cases during the same period suggested that 19.71% of term infants developed hypophosphatemia within three days after birth. This study was to create a nomogram to predict early hypophosphatemia in term infants and to provide evidence for the early detection and treatment of these neonates.

## Methods

### Study object

All neonates hospitalized in the neonatal unit between December 2016 to June 2018 were included in this retrospective study. The serum phosphorus of newborns was tested within three days of birth.

Term infants with serum phosphorus below 1.6 were classified as the hypophosphatemia group (HP group), and those with blood phosphorus above 1.6 were classified as non-hypophosphatemia group (NHP group).

### Inclusion and exclusion criteria

Inclusion criteria: (a). infants who were measured serum phosphorus within three days of birth, (b). All infants are hospitalized in the Neonatal Center of Shunyi Maternal and Children’s Hospital of Beijing Children’s Hospital.

Exclusion criteria: (a). No information available, (b). Tested the serum levels of phosphate over three days for the first time, (c). premature babies, (d). Congenital malformations or dysplasia of the thymus, thyroid, and parathyroid glands in newborns, (e). Without consent.

### Data collection

**Fig. 1:**
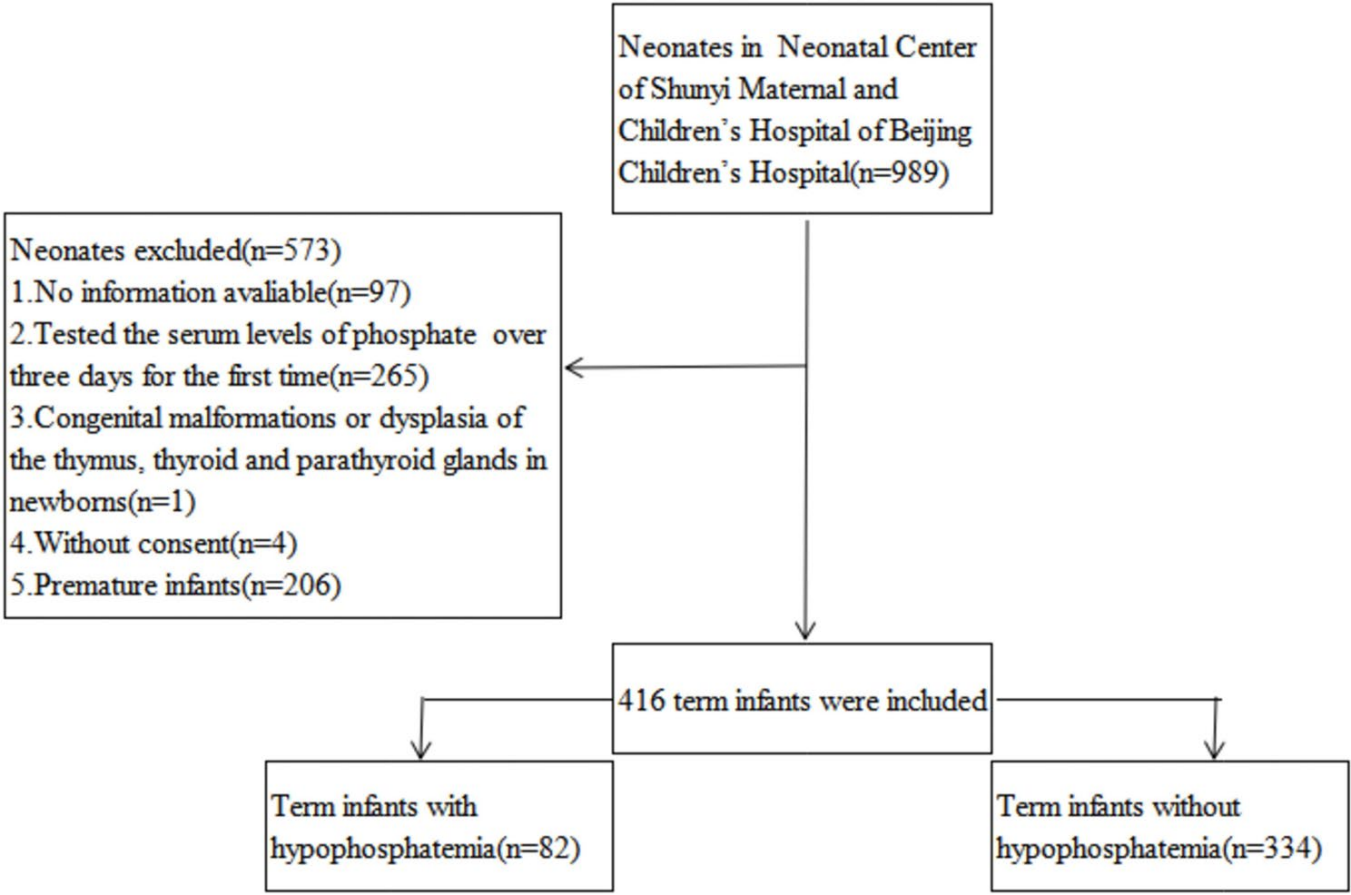
Flow chart for patient selection

We studied 989 patients and collected data from our hospital’s electronic medical record system, including information on patients and their mothers who met the inclusion criteria. A total of 416 patients were included in our study (shown in Fig. 1).

Mothers with gestational diabetes mellitus and hypertension during pregnancy were included in the study, as well as those with intrauterine infection, placental abruption, treated with magnesium sulfate before delivery, and third-degree meconium-stained amniotic fluid. Neonatal-perinatal characteristics include forceps delivery, gender, delivery mode, twin pregnancy, premature rupture of membranes (PROM), gestational age, small for gestational age (SGA), birth weight, neonatal asphyxia, and the times of pregnancy and delivery. The serum biochemical markers of newborns within three days after birth included vitamin D, calcium, magnesium, and alkaline phosphatase (ALP) concentrations.

### Statistical analysis

We used SPSS 26 software (IBM Corp., Armonk, NY, USA) for data analysis and R software (version 4.2.3 R Foundation for Statistical Computing, Vienna, Austria) for nomogram construction.

We used the Student t-test or the Mann-Whitney U test to evaluate continuous data, which was then displayed as mean ± standard deviation (SD) or median and interquartile range. As an alternative, chi-squared or Fisher’s exact tests were used to assess categorical data expressed as numbers (%). We used multivariable logistic regression to identify independent risk factors of hypophosphatemia in term infants based on variables with P-values less than 0.15 in univariable analyses. In the following step, a nomogram was developed based on the independent risk factors extracted from the multivariable logistic regression analysis. We evaluated the predictive ability and performance of the model using the C-index, receiver operating characteristic (ROC) curve, calibration curve, and decision curve analysis (DCA). To evaluate the predictive power of the nomogram and its discriminative ability, we calculated the C-index and the area under the ROC curve (AUC). A higher C-index indicates better accuracy of the nomogram, with values between 0.5 and 1.0. The calibration curve was used to evaluate the relationship between actual diagnoses and predicted probabilities of hypophosphatemia in term infants. Based on the net benefit and threshold probabilities, the DCA was also used to assess the clinical usefulness of the nomogram. A P value of less than 0.05 was considered statistically significant.

## Results

### Patient baseline data

A total of 989 inpatients were tested for blood biochemistry within three days after birth during the period studied. Among which, 573 patients were excluded according to exclusion criteria. It included 82 HP cases and 334 NHP cases in this study. Table 1 displays the fundamental data of the samples, including the mother’s and baby’s perinatal features and the blood biochemical indicators.

**Table 1:** Comparison of clinical features of 416 term infants

	HP(*n* = 82)	NHP(*n* = 334)	X^2^/M	P
Maternal diabetes, n(%)	8(9.8%)	6(1.8%)	10.495	0.001
Hypertensive disorders of pregnancy, n(%)	1(1.2%)	4(1.2%)		1
Treat with magnesium sulphate	1(1.2%)	3(0.9%)		0.586
Intrauterine infection, n(%)	7(8.5%)	16(4.8%)	1.124	0.289
Placental abruption, n(%)	3(3.7%)	2(0.6%)		0.055
Meconium-stained amniotic fluid, n(%)	1(1.4%)	11(3.3%)	0.406	0.524
Forceps delivery, n(%)	9(11%)	34(10.2%)	0.045	0.832
Gender, male, n(%)	55(67.1%)	160(47.9%)	9.688	0.002
Delivery mode, cesarean delivery, n(%)	36(43.9%)	84(25.1%)	11.28	0.001
Neonatal asphyxia, n(%)	5(6.1%)	17(5.1%)	0.008	0.928
Twin pregnancy, n(%)	2(2.4%)	2(0.6%)		0.176
Small for Gestational Age, n(%)	20(24.4%)	24(7.2%)	20.604	<0.001
Birth weight(g)	3355(2667.5,3695)	3425(3090,3720)	11616.5	0.033
Gestational age(week), n(%)	39.4(38.0,40.2)	39.4(38.6,40.2)	13396.5	0.76
Maternal age(year)	28.5(27,31)	28(27,31)	13471.5	0.762
The time of pregnancy	1(1,2)	1(1,2)	12978.5	0.412
The time of delivery	1(1,1)	1(1,1)	13,064	0.347
PROM(h)	0(0,0)	0(0,6.75)	12,117	0.032
25-VitD3(ng/ml)	13.3(9.6,18.225	13.95(10.15,18.5)	13,181	0.599
Calcium(mmol/L)	2.205(2.108,2.38)	2.235(2.088,2.35)	13044.5	0.505
Magesium(mmol/L)	0.765(0.71,0.84)	0.81(0.74,0.88)	10963.5	0.005
ALP(U/L)	123(101,156)	120(102,144)	13,363	0.734

*Abbreviations* HP term infants with hypophosphatemia, PROM premature rupture of membrane, ALP alkaline phosphatase

*A Chi-square test, Fisher’s exact test, Mann-Whitney U test, or Student t-test was used to calculate the *P* value

### Univariate and multivariate analysis

Between the HP and NHP groups, a comparison of serum biochemical marker variables, as well as maternal and neonatal-perinatal features, was carried out (Table 2). There were significant differences in maternal diabetes (*P* = 0.001), placental abruption (*P* = 0.046), male (*P* = 0.002), cesarean delivery (*P* = 0.001), birth weight (*P* = 0.001) and SGA (*P* < 0.001) by univariate analysis. All the risk factors were analyzed in a multivariable logistic regression based on the *P* < 0.15 threshold to identify independent risk factors for HP. According to the multivariate analysis, maternal diabetes (OR = 4.994, 95%CI = 1.577–15.809, *P* = 0.006), male (OR = 2.331, 95% CI = 1.355–4.011, *P* = 0.002), cesarean delivery (OR = 2.142, 95% CI = 1.255–3.657, *P* = 0.005) were independent risk factors for HP (Table 2). Magnesium (OR = 0.07, 95% CI = 0.006–0.827, *P* = 0.035) and birth weight (OR = 0.999, 95% CI = 0.999–1, *P* < 0.001) were independent protective factors for HP.

**Table 2:** Univariate and multivariate binary regression analysis of predictors of HP

	Univariate analysis			Multivariate analysis			
	*p* Value	OR(95%CI)		β	Wald	*p* Value	OR(95%CI)
Maternal diabetes	0.001	5.91(1.991–17.545)		1.608	7.482	0.006	4.994(1.577–15.809)
Hypertensive disorders of pregnancy	0.987	1.019(0.112–9.236)					
Treat with magnesium sulphate	0.79	1.362(0.14-13.266)					
Intrauterine infection	0.19	1.855(0.737–4.669)					
Placental abruption	0.046	6.304(1.036–38.363)		0.624	0.381	0.537	1.867(0.257–13.533)
Meconium-stained amniotic fluid	0.335	0.363(0.046–2.849)					
Forceps delivery	0.832	1.088(0.5-2.368)					
Male	0.002	2.215(1.333–3.682)		0.846	9.346	0.002	2.331(1.355–4.011)
Cesarean delivery	0.001	2.329(1.411–3.845)		0.762	7.792	0.005	2.142(1.255–3.657)
Neonatal asphyxia	0.715	1.2119(0.433–3.384)					
Twin pregnancy	0.158	0.241(0.033–1.737)					
Small for Gestational Age	<0.001	4.167(2.169,8.006)					
Birth weight(g)	0.001	0.999(0.999-1.0)		-0.001	12.499	<0.001	0.999(0.999-1)
Gestational age(week)	0.610	0.954(0.796–1.144)					
Maternal age(year)	0.954	0.998(0.933–1.068)					
The time of pregnancy	0.322	0.877(0.677–1.136)					
The time of delivery	0.316	0.723(0.383–1.363)					
PROM(h)	0.143	0.981(0.956–1.006)		-0.14	1.17	0.279	0.986(0.96–1.012)
25-VitD3(ng/ml)	0.992	1(0.963–1.038)					
Calcium(mmol/L)	0.221	2.1(0.640–6.891)					
Magesium(mmol/L)	0.081	0.119(0.011–1.298)		-2.658	4.455	0.035	0.07(0.006–0.827)
ALP(U/L)	0.486	1.002(0.996–1.008)					

*Abbreviations* OR, odds ratio; CI, confidence interval

Estimated odds ratios based on logistic regression. Significant variables were selected using the “Enter” logistic regression model. According to the multivariate analysis, the risk of HP with maternal diabetes, male, cesarean delivery was 4.994, 2.331, 2.142 times increased respectively. The likelihood of hypophosphatemia rose by 93% for every 1 mmol/L decrease in serum magnesium. Meanwhile, incidence of hypophosphatemia rose by 0.1% for every 1 gram decrease in body weight

### Development and validation of a nomogram for HP

**Fig. 2:**
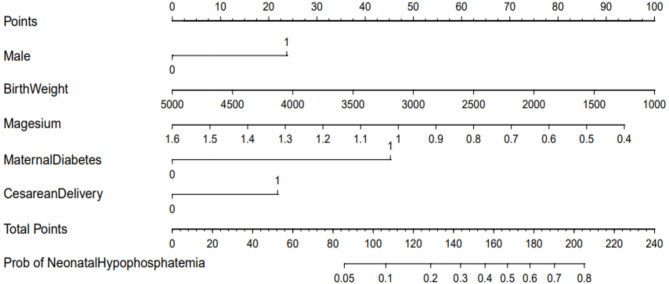
Nomogram for predicting HP risk. A point scale from 0 to 100 was used to score each variable, then the sum of the scores was calculated. The risk of hypophosphatemia in term infants can be predicted by looking at the total points axis. For magesium and birth weight, the units are mmol/L and gram, respectively.For other variables, 0 = no and 1 = yes

Based on the independent risk factors obtained from multivariable logistic regression analyses, we constructed a nomogram to predict hypophosphatemia in term infants (shown in Fig. 2). We selected gestational age rather than SGA to be included in the multivariable logistic regression analysis and nomogram to illustrate the relationship between gestational age and hypophosphatemia in detail.

A point scale from 0 to 100 was used to score each variable, then the sum of the scores was calculated. The risk of hypophosphatemia can be predicted by looking at the total points axis. For magesium and birth weight, the units are mmol/L and gram, respectively. For other variables, 0 = no and 1 = yes

**Fig. 3:**
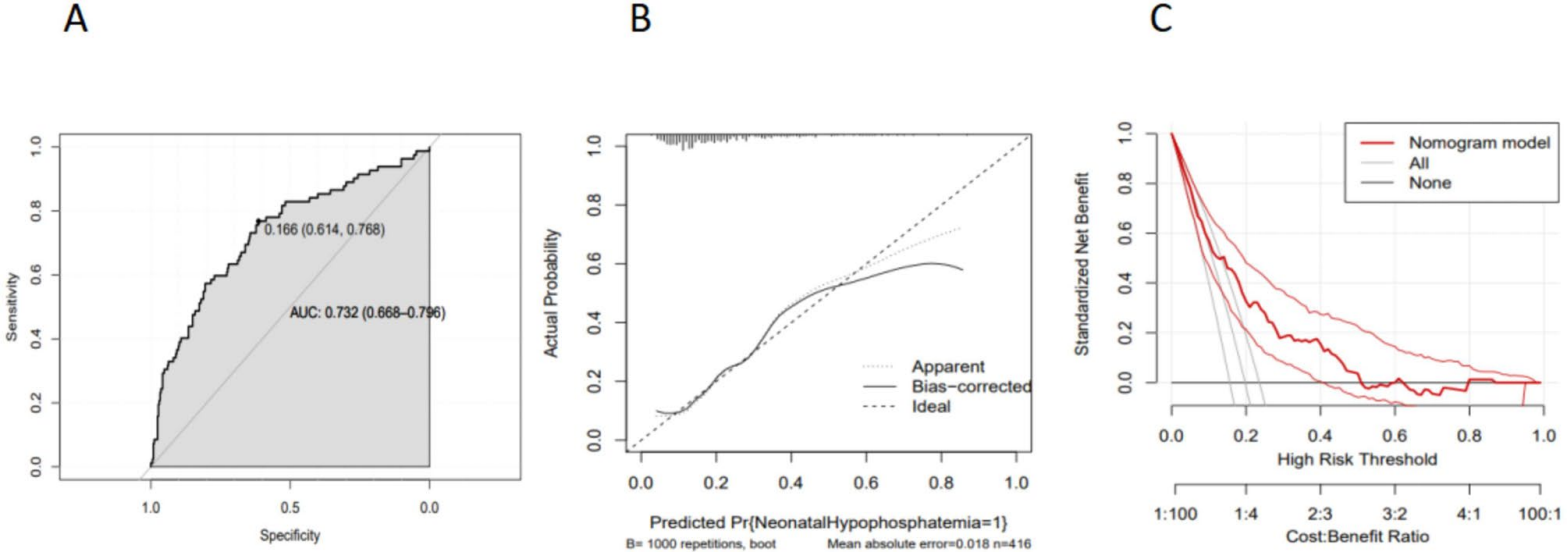
Evaluation of the nomogram model. (**A**) The ROC analysis for the predictive model.The model demonstrated high predictive accuracy and discrimination, with a C- index of 0.732 (95%CI = 0.668–0.796) and an AUC of 0.732. (**B**) The calibration curve for the nomogram.It indicated good consistency between the actual diagnosed early hypophosphatemia in term infants and the predicted probability (bootstrap resampling was 1000 times).(**C**) Decision curve analysis for nomogram of early hypophosphatemia in term infants. The nomogram DCA also indicated that the model could be an excellent tool for predicting early HP in term infants

To show the relation between gestational age, birth weight and hypophosphatemia accurately, we choosed gestational age or birth weight instead of SGA to enter the multivariable logistic regression analysis. A total score was calculated to assess the probability of hypophosphatemia in term infants by adding individual scores of each predictor in the nomogram. Using this method, the risks of HP were evaluated accurately, and serum phosphorus was monitored appropriately. The model demonstrated high predictive accuracy and discrimination, with a C- index of 0.732 (95%CI = 0.668–0.796) and an AUC of 0.732 (shown in Fig. 3A). Moreover, the calibration curve of this nomogram demonstrated fine consistency between the predicted probability and the actual diagnosis of hypophosphatemia in term infants(shown in Fig. 3B). The nomogram DCA also indicated that the model could be an excellent tool for predicting early HP in term infants (shown in Fig. 3C).

**Fig. 4:**
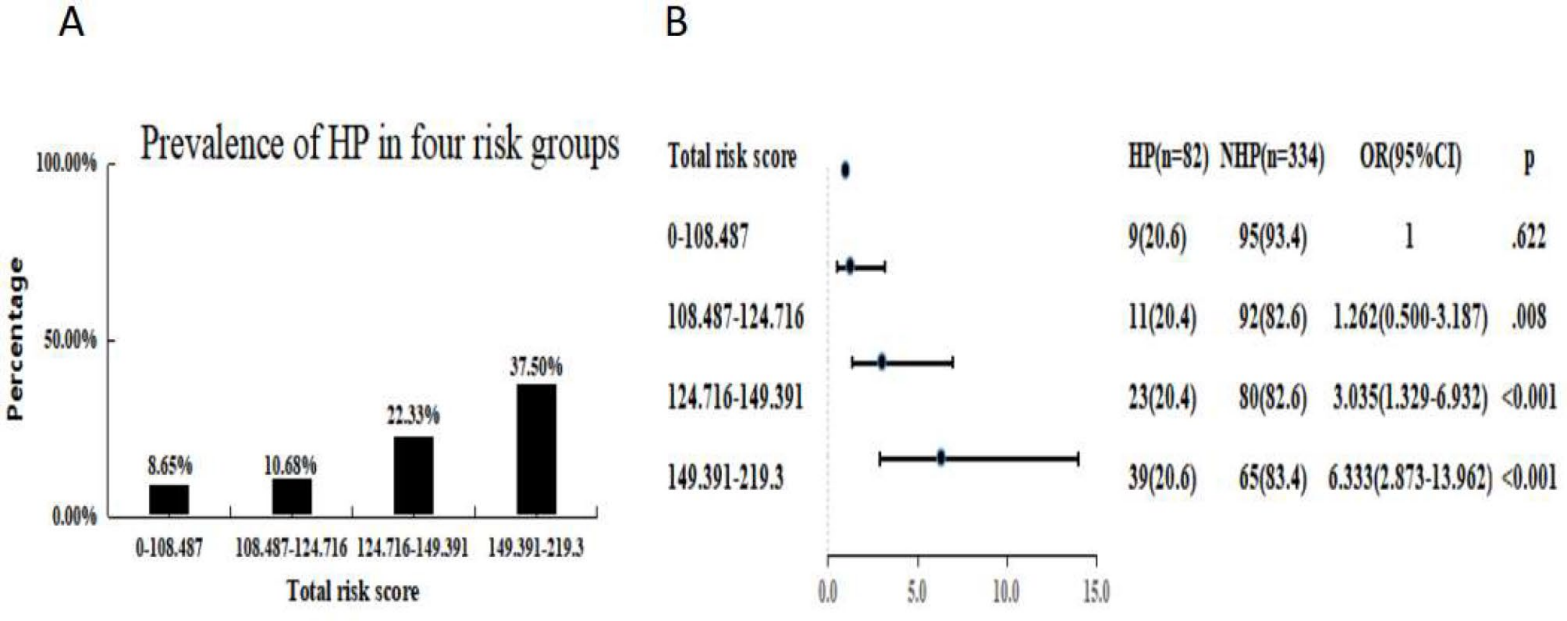
Association between the total points of the nomogram and early hypophosphatemia in term infants. (**A**) The incidence of hypophosphatemia in these quartile-based groups was 8.65%, 10.68%, 22.33%, and 37.5%, respectively. (**B**) participants in quartile three (total points: 124.716-149.391) and quartile four (total points: 149.391–219.3) had a higher risk of HP than those in the lower quartiles (total points: 0-108.487). The odds ratios were 3.035(95%CI: 1.329–6.932) and 6.333 (95%CI: 2.873–13.962) respectively

A quartile-based division of the total points was then conducted, and the risk of hypophosphatemia in term infants was compared. According to Fig. 4A, the incidence of hypophosphatemia in these quartile-based groups was 8.65%, 10.68%, 22.33%, and 37.5%, respectively. Figure 4B shows that participants in quartile three (total points: 124.716-149.391) and quartile four (total points: 149.391–219.3) had a higher risk of HP than those in the lower quartiles (total points: 0-108.487). The odds ratios were 3.035(95%CI: 1.329–6.932) and 6.333 (95%CI: 2.873–13.962) respectively

## Discussion

This study analyzed 416 term infants and developed and validated a predictive clinical nomogram for hypophosphatemia. The rate of hypophosphatemia within three days in term infants was 19.71% according to our findings. The nomogram identified five key related factors such as male, birth weight, magnesium, maternal diabetes, and cesarean delivery

Our study showed male to predict early hypophosphatemia (≤ 3 days). Male were found to have 2.331 times (p-value 0.002, 95%CI 1.355–4.011) higher incidence of hypophosphatemia in term infants compared to female according to our research. It maybe because the transplacental transfer of estrogen is interrupted and lack of self-secretion in male newborns after delivery can lead to elevated parathyroid hormone [22]. Meanwhile, the parathyroid hormone can inhibit the re-absorption of phosphorus in proximal tubules and distal tubules, promote phosphorus metabolism, and increase urine phosphorus

ICU patients are at high risk of depletion of magnesium and phosphate [23]. Anne-Louise Hother et al. reported that serum magnesium and phosphorus levels are positively correlated in children admitted with acute malnutrition. Both serum phosphate and serum magnesium correlated positively with serum albumin [24, 25]. In our investigation, hypophosphatemia decreased as serum magnesium levels increased (p-value 0.035, OR 0.07, 95%CI 0.006–00.827). Which means the likelihood of hypophosphatemia rose by 93% for every 1 mmol/L decrease in serum magnesium. Low serum albumin could be one possible explanation for this trend

Early hypophosphatemia in newborns could be precipitated by several factors, with extremely low birth weight infants having the highest prognostic value. This study showed that the birth weight gain and the incidence of hypophosphatemia in term infants decreased within three days (p-value < 0.001, OR0.999, 95%CI 0.999-1). Which means the likelihood of hypophosphatemia rose by 0.1% for every 1 gram decrease in body weight. According to a survey by Heidi Al-Wassia et al., newborns with birth weight below 1500 g were more susceptible to neonatal hypophosphatemia [26]. Approximately 94% of newborns with birth weight under 1,000 g had hypophosphatemia among them [26]. Tariq et al. reported that there was persistent hypercalcemia in very low birth weight newborns, while serum phosphate levels were consistently lower than the normal range (4–8 mg/dl) [26–29]. In recent years, early nutritional management of small and premature infants has become increasingly popular due to its beneficial effects on the nervous system [29]. However, this nutritional strategy makes them more prone to electrolyte disturbances, including hypophosphatemia. The carbohydrates and amino acids that are consumed increase the serum insulin level, stimulating the entry of phosphorus and potassium into cells. As a result, plasma concentrations of phosphorus and potassium are reduced. Meanwhile, newborns with low birth weight require sufficient protein and energy to promote tissue anabolism, positive nitrogen balance, and somatic cell growth. However, bioaccumulation of phosphate in the low birth weight babies is relatively insufficient. So, babies with low birth may develop severe hypophosphatemia three to four days after receiving intravenous nutrition

In line with previous studies, our data showed that term infants with maternal gestational diabetes mellitus were more likely to suffer from hypophosphatemia. It could predict early hypophosphatemia in three days. Currently, at least three mechanisms have been identified as the main reasons for maternal diabetes associated with hypophosphatemia. Firstly, the fetus of mothers with maternal diabetes has chronic hypoxia phenomenon in the uterus. Which may promote the secretion of calcitonin, then lead to hypophosphatemia and hypocalcemia of term infants in the early postnatal period. At the same time, when pregnant mothers have maternal gestational diabetes mellitus or abnormal blood sugar, it is possible to deliver macrosomia or low birth weight newborns [30] with increased demand for phosphorus. Studies show that maternal gestational diabetes increases the risk of placental inflammation and that transplacental phosphate transport is blocked in placental inflammation. These factors might explain the tendency of hypophosphatemia in term infants with diabetic mothers in the early postnatal period (≤ 3 days)

　In addition, cesarean delivery is vital in guiding hypophosphatemia in term infants. In our study, we showed that cesarean delivery would increase the incidence of hypophosphatemia in term infants. The probability of hypophosphatemia in term infants delivered by cesarean section within three days after birth is 2.142 times that of term infants delivered naturally (p-value 0.005, 95%CI 1.255–3.657). In term and preterm infants, the American College of Obstetricians and Gynecologists (ACOG) recommended delaying umbilical cord clamping for at least 30–60 s after birth [31, 32]. However, it is not actively implemented in cesarean section due to the fear of excessive maternal blood loss and neonatal jaundice. This might explain why cesarean section could cause neonatal anemia [33]. Few studies took the effect of different delivery methods on delayed cord clamping (DCC) into account now. In cesarean sections, early cord clamping (ECC) is often implemented rather than DCC, which may indicate that cesarean sections result in less placental transfusion. During this time, phosphate transfer through the umbilical cord decreased. As a result, term infants delivered by cesarean section are more likely to have hypophosphatemia than term infants delivered vaginally

We developed a predictive nomogram for hypophosphatemia based on potential risk factors such as male, low birth weight, low concentration of serum magnesium, maternal diabetes, and cesarean delivery. The nomogram could be helpful in assessing the risk of hypophosphatemia in term infants within three days. As the constructed model predicted, patients with low birth weight are at high risk of hypophosphatemia. In addition, hypophosphatemia is less likely to occur in patients with higher serum magnesium levels. A nomogram for predicting hypophosphatemia in term infants was successfully developed and validated in our study. The nomogram is visual and easy to understand. However, we conducted our study retrospectively, which may have resulted in inherent selection bias. Furthermore, since serum phosphorus was not measured simultaneously, the incidence of early hypophosphatemia in term infants may be underestimated. What’s more, research on neonates during their diuretic phase is limited due to our study’s focus on infants less than three days. which may potentially lead to lower levels in conditions like respiratory distress syndrome, transient tachypnea of the newborn, or sepsis. We will take further investigation into this aspect

## Conclusion

Our results demonstrate the feasibility of developing and validating a nomogram model for individualized prediction of hypophosphatemia in term infants within three days based on perinatal variables. According to the nomogram model, term infants with high predictive values should have serum phosphate levels detected as soon as possible to correct hypophosphatemia early and reduce the incidence of potential complications and metabolic bone diseases

## Data Availability

The data contributing to this article may be made available upon request by sending an e-mail to the first author.

## Abbreviations

ATP: Adenosine triphosphate
HP: Group term infants with hypophosphatemia
NHP: Group term infants without hypophosphatemia
DCA: Decision curve analysis
ICUs: Intensive care units
PROM: Premature rupture of membranes
SGA: Small for gestational age
ALP: Alkaline phosphatase
ROC: Receiver operating characteristic
AUC: Area under the ROC curve
ACOG: American College of Obstetricians and Gynecologists
DCC: Delayed cord clamping
ECC: Early cord clamping

## References

[1] Geerse DA, Bindels AJ, Kuiper MA, Roos AN, Spronk PE, Schultz MJ (2010). Treatment of hypophosphatemia in the intensive care unit: a review. Crit Care.

[2] Amanzadeh J, Reilly RF (2006). Hypophosphatemia: an evidence-based approach to its clinical consequences and management. Nat Clin Pract Nephrol.

[3] Florenzano P, Cipriani C, Roszko KL, Fukumoto S, Collins MT, Minisola S, Pepe J (2020). Approach to patients with hypophosphataemia. Lancet Diabetes Endocrinol.

[4] Gaasbeek A, Meinders AE (2005). Hypophosphatemia: an update on its etiology and treatment. Am J Med.

[5] Imel EA, Econs MJ (2012). Approach to the hypophosphatemic patient. J Clin Endocrinol Metab.

[6] Cioffi I, Ponzo V, Pellegrini M, Evangelista A, Bioletto F, Ciccone G, Pasanisi F, Ghigo E, Bo S (2021). The incidence of the refeeding syndrome. A systematic review and meta-analyses of literature. Clin Nutr.

[7] Imel EA (2021). Congenital conditions of Hypophosphatemia in Children. Calcif Tissue Int.

[8] Choi HS, Kwon A, Chae HW, Suh J, Kim DH, Kim HS (2018). Respiratory failure in a diabetic ketoacidosis patient with severe hypophosphatemia. Ann Pediatr Endocrinol Metab.

[9] Naik VM, Saifuddin MS, Nair AS, Rayani BK (2019). Acute onset quadriparesis following oesophagectomy due to isolated hypophosphataemia. Indian J Anaesth.

[10] Sprung J, Weingarten TN (2014). Severe hypophosphatemia: a rare cause of postoperative muscle weakness. J Clin Anesth.

[11] Bosman A, van den Beld AW, Feelders RA, Zillikens MC (2021). Cortisol and phosphate homeostasis: Cushing’s syndrome is Associated with reversible hypophosphatemia. Front Endocrinol (Lausanne).

[12] Megapanou E, Florentin M, Milionis H, Elisaf M, Liamis G (2020). Drug-Induced Hypophosphatemia: current insights. Drug Saf.

[13] Bradford CV, Cober MP, Miller JL (2021). Refeeding syndrome in the neonatal Intensive Care Unit. J Pediatr Pharmacol Ther.

[14] Blanc S, Vasileva T, Tume LN, Baudin F, Chessel Ford C, Chaparro Jotterand C, Valla FV (2022). Incidence of Refeeding Syndrome in critically Ill Children with Nutritional Support. Front Pediatr.

[15] Veldscholte K, Veen MAN, Eveleens RD, de Jonge RCJ, Vanhorebeek I, Gunst J, Casaer MP, Wouters PJ, Guerra GG, Van den Berghe G (2022). Early hypophosphatemia in critically ill children and the effect of parenteral nutrition: a secondary analysis of the PEPaNIC RCT. Clin Nutr.

[16] Garcia Martin A, Varsavsky M, Cortes Berdonces M, Avila Rubio V, Alhambra Exposito MR, Novo Rodriguez C, Rozas Moreno P, Romero Munoz M, Jodar Gimeno E, Rodriguez Ortega P (2020). Phosphate disorders and clinical management of hypophosphatemia and hyperphosphatemia. Endocrinol Diabetes Nutr (Engl Ed).

[17] Pistolesi V, Zeppilli L, Fiaccadori E, Regolisti G, Tritapepe L, Morabito S (2019). Hypophosphatemia in critically ill patients with acute kidney injury on renal replacement therapies. J Nephrol.

[18] Statlender L, Raphaeli O, Bendavid I, Hellerman M, Kagan I, Fishman G, Singer P. Correlations between First 72 h Hypophosphatemia, Energy Deficit, Length of Ventilation, and Mortality-A Retrospective Cohort Study. Nutrients 2022, 14(7). 10.3390/nu14071332 PMC9002762 35405945

[19] Liu B, Cheng Y, Shen F, Wang Y, Wu Y, Yao L, Liu Y, Gou X (2018). [Hypophosphatemia is associated with poor prognosis of critically ill patients: a meta-analysis of 1 555 patients]. Zhonghua Wei Zhong Bing Ji Jiu Yi Xue.

[20] Abrams SA (2007). In utero physiology: role in nutrient delivery and fetal development for calcium, phosphorus, and vitamin D. Am J Clin Nutr.

[21] Fenton TR, Lyon AW, Rose MS (2011). Cord blood calcium, phosphate, magnesium, and alkaline phosphatase gestational age-specific reference intervals for preterm infants. BMC Pediatr.

[22] Chacham S, Pasi R, Chegondi M, Ahmad N, Mohanty SB (2020). Metabolic bone disease in premature neonates: an unmet challenge. J Clin Res Pediatr Endocrinol.

[23] Vesterlund GK, Thorsen-Meyer HC, Moller MH, Brunak S, Strom T, Perner A, Kaas-Hansen BS (2023). Abnormal serum levels of magnesium, phosphate, and zinc in ICU patients-Characteristics, management, and outcomes: the WhyTrace cohort study. Acta Anaesthesiol Scand.

[24] Hother A-L, Girma T, Rytter MJH, Abdissa A, Ritz C, Mølgaard C, Michaelsen KF, Briend A, Friis H, Kæstel P. Serum phosphate and magnesium in children recovering from severe acute undernutrition in Ethiopia: an observational study. BMC Pediatr 2016, 16(1). 10.1186/s12887-016-0712-9 PMC5097423 27814707

[25] Kroll MH, Elin RJ (1985). Relationships between magnesium and protein concentrations in serum. Clin Chem.

[26] Al-Wassia H, Lyon AW, Rose SM, Sauve RS, Fenton TR (2019). Hypophosphatemia is prevalent among Preterm infants less than 1,500 Grams. Am J Perinatol.

[27] Brener Dik PH, Galletti MF, Fernandez Jonusas SA, Alonso G, Mariani GL, Fustinana CA (2015). Early hypophosphatemia in preterm infants receiving aggressive parenteral nutrition. J Perinatol.

[28] Early. hypophosphatemia in very low birth weight preterm infants. 10.17219/acem/70081 29790700

[29] Cubillos Celis MP, Mena Nannig P (2018). [Hypophosphatemia in preterm infants: a bimodal disorder]. Rev Chil Pediatr.

[30] Civantos Modino S, Duran Martinez M, Flandez Gonzalez B, Martell Claros N, Fernandez Perez C, Navea Aguilera C, Merino Viveros M, de Guijarro G, de Pavon I, Monereo Megias S (2019). Implication of gestational diabetes treatment on maternal weight gain and low neonatal weight: a large retrospective cohort study. Nutr Hosp.

[31] Shao H, Gao S, Lu Q, Zhao X, Hua Y, Wang X (2021). Effects of delayed cord clamping on neonatal jaundice, phototherapy and early hematological status in term cesarean section. Ital J Pediatr.

[32] American College of O, Gynecologists’ Committee on Obstetric P (2020). Delayed umbilical cord clamping after birth: ACOG Committee Opinion, Number 814. Obstet Gynecol.

[33] Shibata T, Nakago S, Nishikawa S, Fukuoka Y, Iizuka N, Kotsuji F (2020). A disadvantage of cesarean section en caul: umbilical velamentous insertion, a risk factor and proposed mechanism of neonatal anemia. J Obstet Gynaecol Res.

